# Pineal Parenchymal Tumors With Intermediate Differentiation to Pineoblastoma: A Transitional Neuroectodermal Tumor of the Pineal Gland

**DOI:** 10.7759/cureus.38495

**Published:** 2023-05-03

**Authors:** Pooja Jha, Samarth Shukla, Sunita Vagha, Sourya Acharya

**Affiliations:** 1 Pathology, Jawaharlal Nehru Medical College, Datta Meghe Institute of Higher Education & Research (DMIHER), Wardha, IND; 2 Medicine, Jawaharlal Nehru Medical College, Datta Meghe Institute of Higher Education & Research (DMIHER), Wardha, IND

**Keywords:** third ventricular tumors, who grade, pineoblastoma, pineal tumor of intermediate differentiation, pineal tumors

## Abstract

Pineal tumors are quite rare and are fairly aggressive tumors seen in young adults and children. These tumors arise from the pineal region or recess from various types of cells in the gland and structures located in close propinquity to the gland. Pineal gland tumors have a heterogeneous spectrum that includes pineal parenchymal tumors (PPTs) and papillary tumors of the pineal region (PTPR). The PPTs are further subclassified into pineocytomas (Grade 1), PPTs of intermediate differentiation (grade 2 or 3), and pineoblastomas (grade 4) based on the World Health Organization (WHO) grades and histopathological features. We discuss the case of an 11-year-old male child who presented with complaints of headache for 15 days, vomiting for seven days, and diplopia for four days. On magnetic resonance imaging (MRI), a soft tissue density lesion was noticed in the posterior third ventricle region. Based on the location and the MRI findings, the differential diagnosis considered were a pineal lesion, a choroid plexus papilloma, or a meningioma. He underwent a right occipital ventriculoperitoneal shunt followed by total excision of the tumor, and the resected specimen was sent for histopathological examination. After pathologic examination, the diagnosis of pineoblastoma (grade 4) with features of a PPT of intermediate differentiation (grades 2-3) was revealed, and the same was confirmed on immunohistochemistry.

## Introduction

The first anatomist to discover pineal tumors was Herophilus (325-280 BC), who is also known as the *Father of Anatomy* as reported by Aelius Galenus, a Greek physician (130-200 AD) [1]. He suggested scientific ways to perform anatomical dissection and also described the gland as a valve or sphincter that regulates the flow of *pneuma* from the third ventricle to the fourth ventricle [1]. Pineal tumors have been an interesting matter of discussion for many decades. The origin of the histopathological classification of pineal tumors and their evolution were initiated after Krabbe, in his research work titled *Histological Studies of the Pineal Body*, used the term *Pinealoma* for the first time [2]. Thereafter, Percival Bayley, Cushing, and Bailey, followed by Russell, Rubenstein, Zulch, Globus, and Dorothy Russel, and Nathan Friedman, provided the basis of the histopathological classification of pineal tumors [3-5]. Anne Jouvet, a neuropathologist from France in 2000, made a significant contribution to the World Health Organization (WHO) classification with ideas on pathological aspects [6].

As we discuss these rare entities, with all the limited information that we have for pineal tumors, we have tried to study various histopathological features, correlating them with radiological and immunohistochemical findings. The pineal parenchymal tumor of intermediate differentiation and pineoblastoma are the topics of interest in our case. The transition of the tumor from grades 2-3 to grade 4 shows the malignant ability tumor possesses and has an influence on the prognosis and therapeutic significance.

## Case presentation

An 11-year-old male child came to the pediatric outpatient department (OPD) at our hospital with the presenting problem of a headache for 15 days, a history of vomiting on and off for seven days, and diplopia for four days. He was well before that, and there was no similar or any other significant history. The general examination was within normal limits. The neurologic examination was within normal limits except for papilledema in both eyes, which was suggestive of raised intracranial pressure.

**Figure 1:**
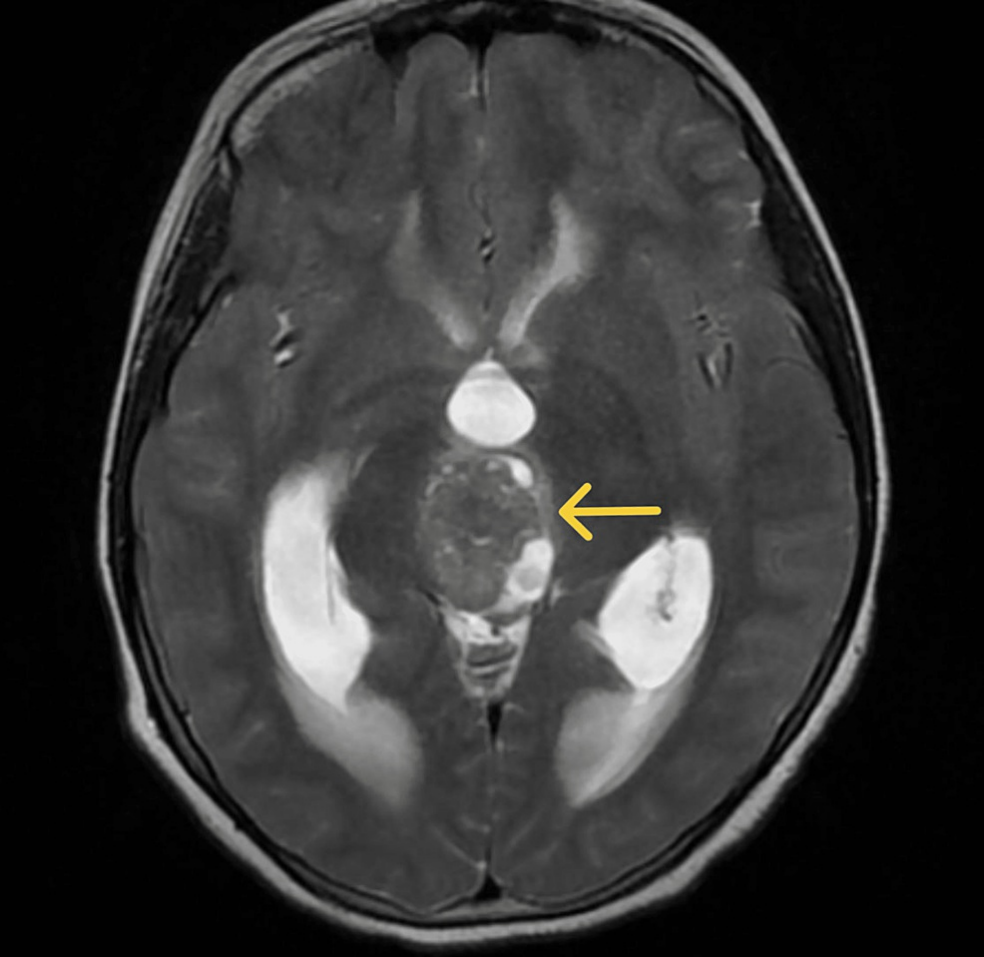
An axial T2 image showing a solitary, lobulated, irregular isointense mass lesion in the posterior third ventricle, with areas of hypointensity along with cystic changes. The mass blocks the third ventricle outflow, causing the resultant hydrocephalus.

**Figure 2:**
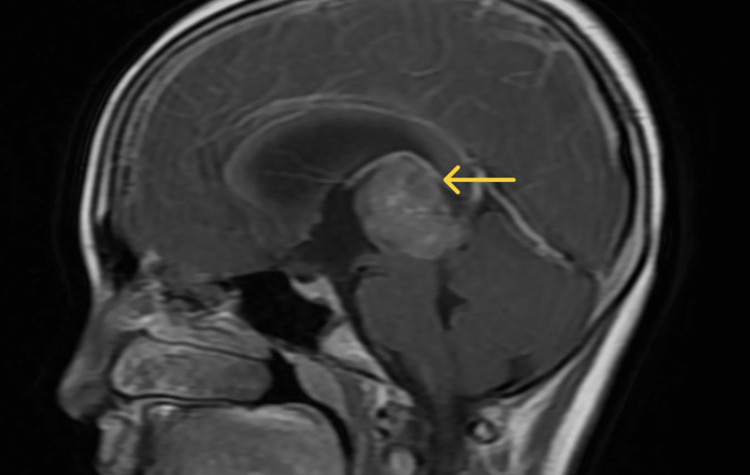
Postcontrast sagittal MRI shows significant heterogenous contrast uptake by the lesion. MRI, magnetic resonance imaging

The patient was admitted to the pediatric ward, and an MRI of the brain with contrast was performed, which revealed an irregularly lobulated soft tissue density lesion exhibiting an intense enhancement in the posterior third ventricle region with a cerebrospinal fluid (CSF) cleft, suggesting it to be a choroid plexus papilloma; along with this, pineal tumors and meningioma were considered as the differentials (Figures 1, 2).

He underwent a right occipital ventriculoperitoneal (VP) shunt the very next day right after all the primary investigations were accomplished. He mentioned relief from headaches postprocedure. Surgery for tumor excision was done three days later. During admission, the complete blood count report was within normal limits. The erythrocyte sedimentation rate was raised, i.e., 26 mm/hour. The patient was managed with antibiotics, analgesics, antacids, antiepileptics, and other supportive measures. The patient was kept for observation for one-week post-surgery and was hemodynamically stable, conscious, and oriented. Hence, he was discharged with the follow-up plan after 15 days in neurosurgery OPD. The final diagnosis was given as pineoblastoma on histopathology.

An MRI brain spectroscopy was also performed that revealed raised levels of lipid lactate and choline with reduced *N*-acetyl-aspartate (NAA) levels. Thereafter, an immediate neurosurgical opinion was taken, and the patient was planned for a right occipital VP shunt. The CSF collected during this pressure was negative for markers of germ cell tumors and was also negative for any malignant cells on cytology. He then underwent midline suboccipital craniotomy and supracerebellar infratentorial excision of the lesion. The resected sample was sent for histopathological examination. On gross examination, the tumor was irregular, grayish yellow in color, soft, friable, and mildly gelatinous with areas of hemorrhage and necrosis.

**Figure 3:**
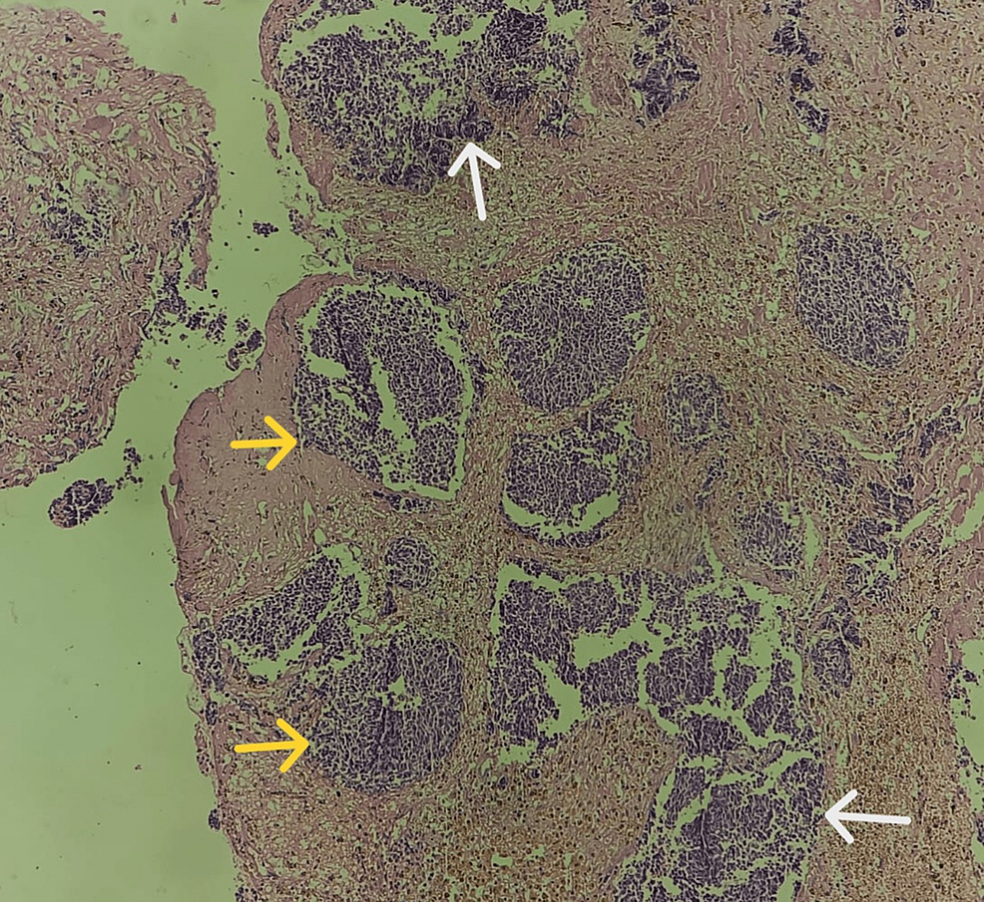
Tumor cells arranged majorly in lobules (yellow arrow) and also in sheets (white arrow) at a few places separated by fibrovascular septa (hematoxylin and eosin stain, 10x view).

**Figure 4:**
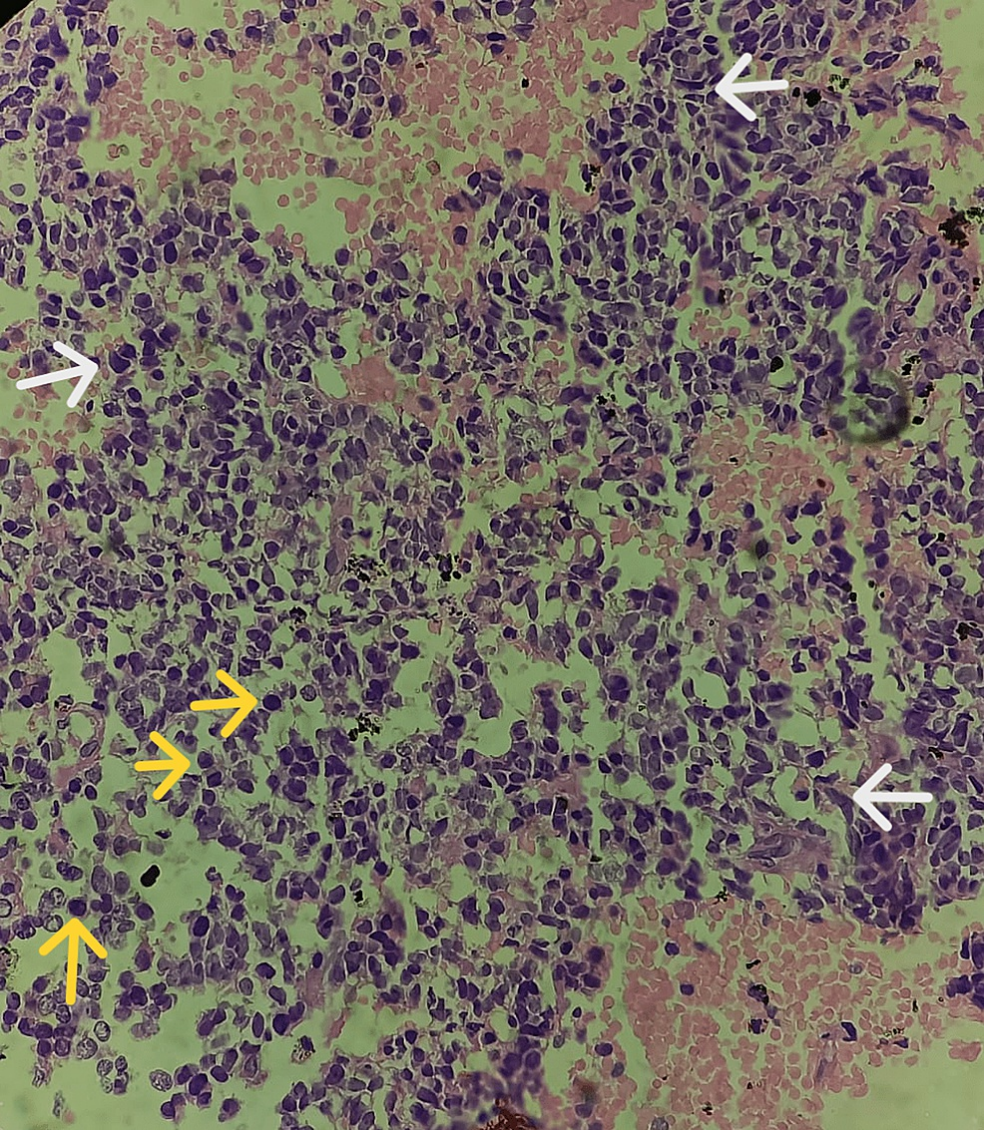
Tumor cells had a typical appearance of round blue cell tumors (yellow arrows), some were angulated in shape, anaplastic in nature with large hyperchromatic nuclei along with a few cells showing nuclear molding, stippled chromatin, inconspicuous nuclei, and very little eosinophilic cytoplasm (white arrows; hematoxylin and eosin stain, 40x view).

**Figure 5:**
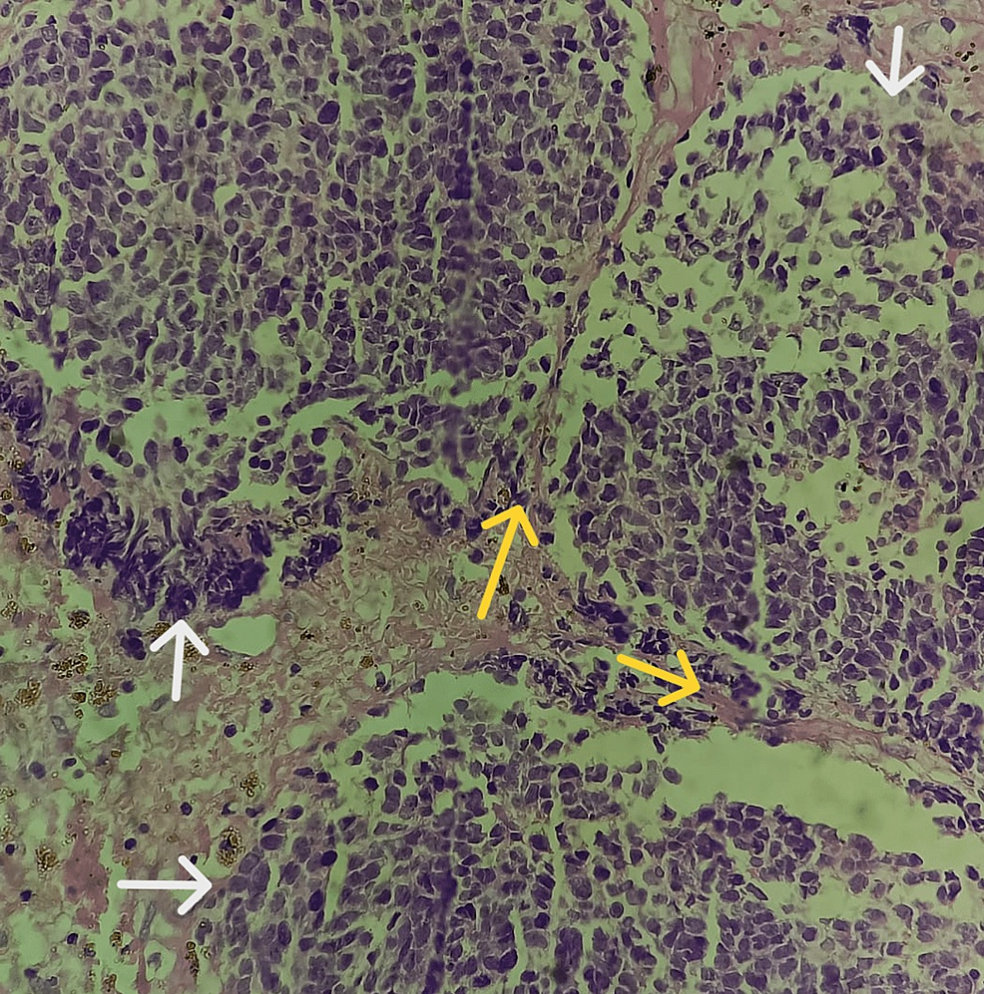
Tumor cells arranged majorly in lobules (white arrows) at a few places separated by fibrovascular septa (yellow arrows; hematoxylin and eosin stain, 40x view).

**Figure 6:**
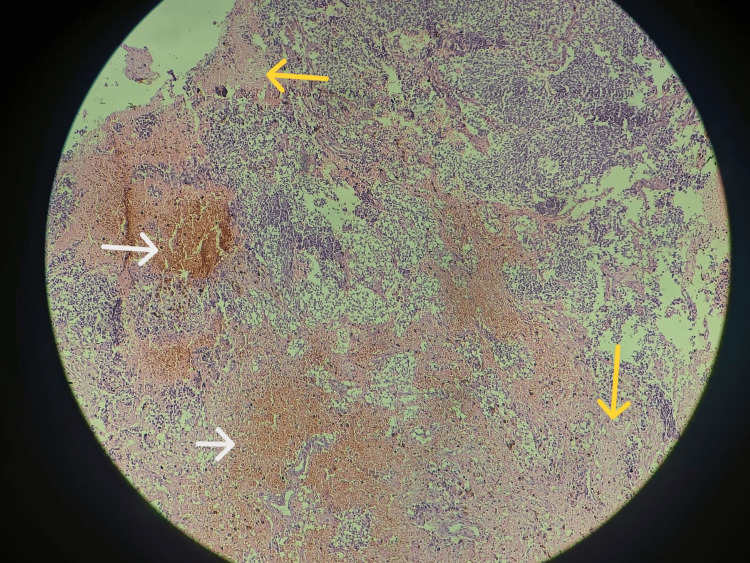
Areas of foci of necrosis (yellow arrows) and extensive hemorrhage (white arrows) were evident along with microvascular proliferation at one or two places (hematoxylin and eosin stain, 10x view).

**Figure 7:**
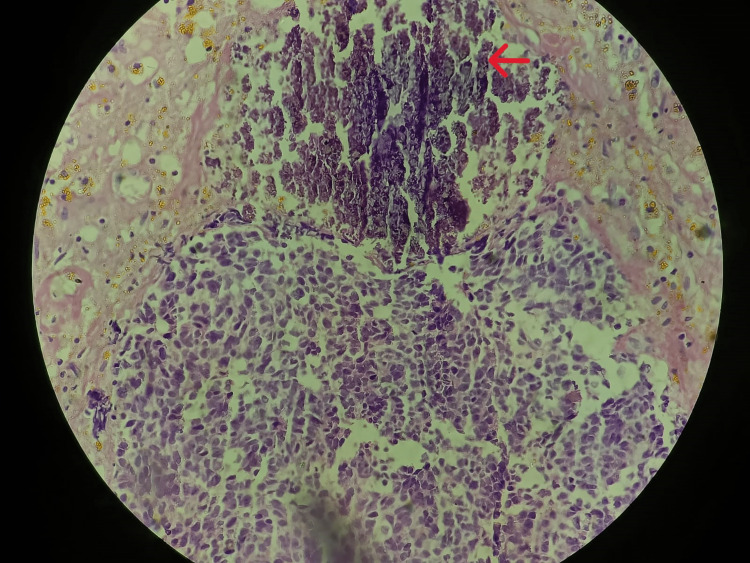
A focus of calcification was also noticed (hematoxylin and eosin stain, 40x view).

**Figure 8:**
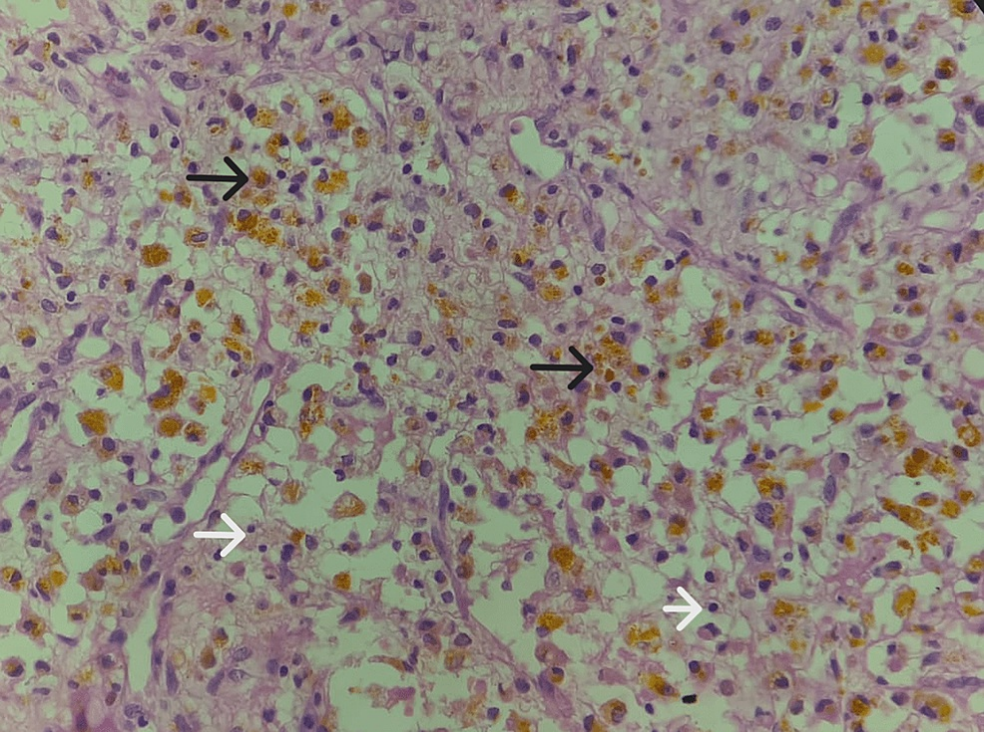
Tumor cells showing the presence of numerous hemosiderin-laden macrophages (black arrows) along with inflammatory cells (white arrows) in the background (hematoxylin and eosin stain, 40x view).

On microscopy, the tumor was unencapsulated with primitive-appearing tumor cells arranged majorly in lobules and also in sheets at a few places separated by fibrovascular septa (Figures 3, 4). The tumor cells had the typical appearance of a round blue cell tumor; some were angulated in shape and anaplastic with large hyperchromatic nuclei, along with a few cells showing nuclear molding, stippled chromatin, inconspicuous nuclei, and very little eosinophilic cytoplasm (Figure 5). No Homer-Wright rosettes were identified. Areas of necrosis and extensive hemorrhage were evident along with microvascular proliferation seen at one or two places (Figure 6). Mitotic activity was sparse. A focus on calcification was also noticed (Figure 7). The tumor also had the presence of numerous hemosiderin-laden macrophages along with inflammatory cells in the background (Figure 8). Although the tumor had very few areas with grade 2 features, most areas showed features of the highly proliferative lesion, i.e., the transition from the pineal tumor of intermediate differentiation (PPTID) to pineoblastoma. All the aforementioned features favored the final diagnosis of pineoblastoma (grade 4).

**Figure 9:**
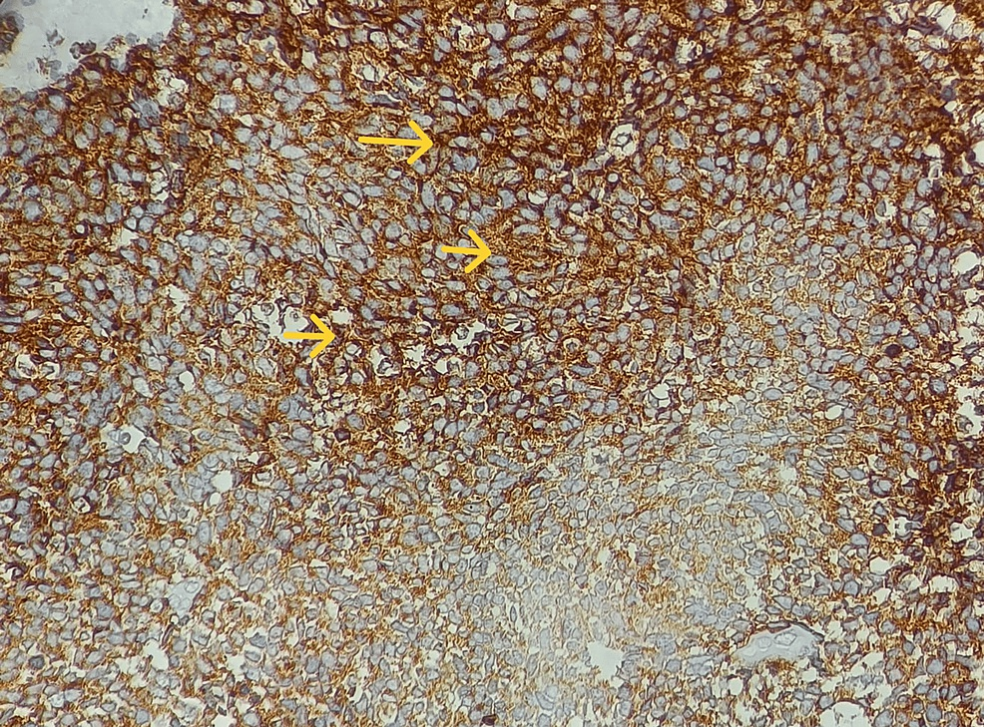
Tumor cells showing strong diffuse cytoplasmic immunoreactivity for synaptophysin (40x view).

**Figure 10:**
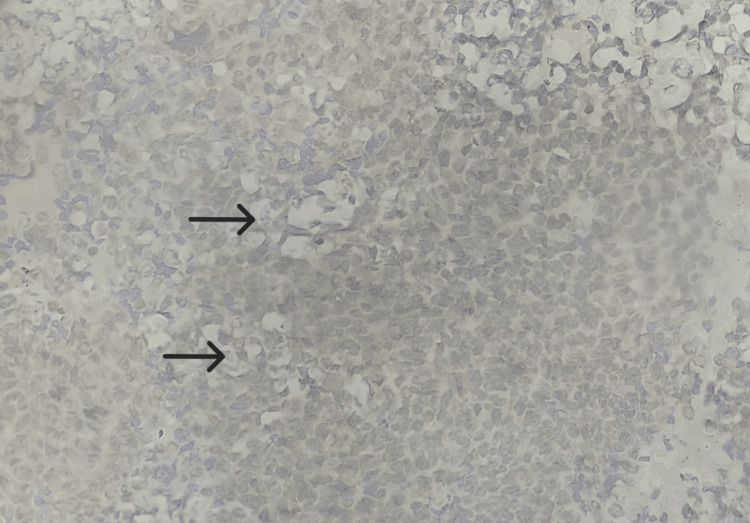
Tumor cells showing negative immunoreactivity for OCT 3/4 (40x view). Tumor cells showing negative immunoreactivity for OCT 3/4 (40x view). OCT 3/4, Octamer-binding transcription factor 3/4

Immunohistochemistry (IHC) of tumor cells showed strong diffuse immunoreactivity for synaptophysin, indicating it to be a neuroendocrine tumor (Figure 9). To check the proportion of proliferation of the tumor, Ki67 was also performed and the proliferation index was reported as more than 30%. Octamer-binding transcription factor 3/4 (OCT 3/4) was also performed to rule out germ cell tumors; it showed negative immunoreactivity (Figure 10). Negative expression was also noticed with glial fibrillary acidic protein (GFAP), which ruled out the possibility of a glioma. Epithelial membrane antigen (EMA) expression was also negative, and thus, meningioma was ruled out. He received both chemotherapy and radiotherapy and was asked to keep up with regular follow-ups. 

 

## Discussion

Pineal tumors are quite rare and account for less than 0.3% of all CNS tumors in young adults and up to 8% in children. It usually occurs in children, the average age at diagnosis is 13 years, and exceptionally rare in adults [6]. Females are observed to be more affected. In our case scenario, the patient is a male child of 11 years of age. The survival rate of pediatric patients is markedly worse than adult patients [6]. It is considered a malignant supratentorial midline primitive neuroectodermal tumor (sPNET) arising from the pineal gland. Earlier, Schild et al. described the four types of pineal parenchymal tumors: pineocytomas, intermediate tumors, mixed tumors, and pineoblastomas [7]. The intermediate type was observed to be an intermediate form between pineocytomas and pineoblastomas. The features of both pineocytomas and pineoblastomas were seen in mixed tumors. In addition to Schild's grading system, Fauchon et al. added another important parameter for analyzing the number of mitoses and their correlation with survival [8]. The two-tiered grading system was used earlier for most of these pineal tumors [9,10]. The 2021 WHO Classification of Tumors of the CNS recognizes five main groups of tumors in the pineal region: pineocytoma, pineal parenchymal tumor of intermediate differentiation, papillary tumor of the pineal region, pineoblastoma and desmoplastic myxoid tumor of the pineal region - SMARCB1-mutant tumor [11]. In 2007, PPTID was considered as a separate entity to assemble a group of tumors between pineoblastomas and pineocytomas according to histological grade [12]. Till now, specific diagnostic criteria for PPTID have not been established. However, the transition from PPTID to pineoblastoma has been noted.

In our case, on MRI brain spectroscopy, raised levels of lipid lactate and choline with reduced NAA levels were noted and a high choline/NAA ratio (>2.5) was taken into account as it is an indicator of a high-grade tumor [13]. Cancer cells produce lactate via glycolysis (the Warburg effect) [14]. The increased levels of lipid lactate also indicate a high-grade tumor as it is a marker of anaerobic metabolism and is observed to be elevated in tumors with necrotic areas [14].

## Conclusions

As such tumors are rare, information regarding these tumors is very limited. There is a constant effort undergoing to have substantial information regarding these tumors, which will help us to define the specific treatment options. We have very few studies and limited data available related to these tumors. Our study adds to the limited information on this topic and also showcases that though it is seen more commonly in children specifically in young females, in our case, the patient is a 11-year-old boy. There are no definite criteria to grade these tumors as yet. For now, pineal tumors are evaluated on the basis of biological and pathological features along with the help of immunohistochemistry, which includes mitosis, Ki 67 proliferation, and neurofilament protein expression.
